# Design of robotic arm for the porcelain bushing in substation

**DOI:** 10.1038/s41598-024-58443-7

**Published:** 2024-04-02

**Authors:** Hao Chen, Wei Han, Weilun Xu, Zongyao Tang, Yini Chen, Peng Xu, Zhaoxing Ma

**Affiliations:** 1 State Grid Jiangsu Electric Power Co. Ltd. Nanjing Power Supply Branch, Nanjing, 211102 China; 2 Electric Power Research Institute of State Grid Henan Electric Power Company, Zhengzhou, 450052 China; 3 State Grid Jiangsu Electric Power Co. Ltd. EHV Branch, Nanjing, 211100 China; 4 State Key Laboratory of Technology and Equipment for Defense against Power System Operational Risks, NARI Group Co., Ltd., Nanjing, China; 5 School of Information and Control Engineering, Qingdao University of Technology, Qingdao, China

**Keywords:** Porcelain, Bushing, Robotic arm, Regime switching function, Automatic orientation, Energy science and technology, Engineering

## Abstract

With the development and the application popularization of artificial intelligence robot technology and 5G technology, a robotic arm is designed and developed for rinsing porcelain bushing in high voltage substation in this paper. Firstly, the components and implementation of robotic arm are presented, subsequently, a circular cleaning structure with a 120-degree split is proposed to rinse the porcelain bushing. Secondly, a two-stage simple and effective method to realize automatic orientation is proposed utilizing photoelectric switches. Moreover, a prototype of robotic arm with control system is developed based on the regime switching function, and the result of edge computing is transmitted by 5G technology. Finally, feasibility and effectiveness of the robotic arm are verified in the Nanjing power grid. The case study manifests that the robotic arm developed by the proposed method in the paper can achieve efficient rinsing and all the corresponding information can be transmitted preciously. The proposed method lays a foundation for wide application of cleaning robot in high voltage substation.

## Introduction

Along with the development of economy and the acceleration of industrialization in China, devices in substation have caught more and more attention of electric engineer in this field^1–3^. Porcelain bushing is a kind of typical insulators widely adopted in substation^4,5^. It is the essential added part of external insulation. As a critical part of power grid, High Voltage (HV) substations play a significant role in the power supply. Therefore, HV should be operated safely on many electrical devices, such as circuit breaker, transformer, current transformer (CT), etc. If the porcelain bushings are contaminated, the corresponding electrical devices will be in the danger condition. Especially, in heavy foggy and other severe weather conditions, the pollution attached to the surface of insulator is inclined to cause flashover, large-scale power failure, or even casualties^6–8^. Consequently, it is urgent to keep porcelain bushing clean lest flashover happens.

The conventional methods of porcelain bushing cleaning require workers to clean it manually. The maintenance workers need to climb the electrical devices or stand on a platform to clean the porcelain bushing with special cleaning cloth or electrical cleaning brush. It is obvious that manual methods are limited to low efficiency and high security risk^9,10^.

In recent years, a wealth of new technologies, such as robotic technology, have promoted the work quality of insulator cleaning in HV substations and transmission lines^11,12^. Fang et al.^13^ developed a type of efficient climbing robot for suspension insulator strings of extra high voltage transmission line. Pu et al.^14^ discussed the electric field analysis and field test of 500 kV insulator detection robot. Griecoa et al.^15^ discussed Internet of Things (IoT) technologies^16,17^ and the application of robot using IoT in industrial plants. Park et al.^18^ proposed a novel cleaning robot system for live-line suspension insulator strings.

Overall, the cleaning of insulators can be mainly divided into two types: cleaning while powered and cleaning while shutdown. Currently, the cleaning of ceramic post insulators in engineering applications mainly relies on manual cleaning methods, including power shutdown manual cleaning, water cleaning while powered, and air blowing cleaning while powered. These methods have the disadvantages of low cleaning efficiency, difficulty, high dependence on personnel for cleaning quality, low consistency of cleaning quality, and difficulty in ensuring the safety of workers. To address these issues, it is necessary to develop an automatic cleaning device for ceramic post insulators that can improve cleaning efficiency while ensuring consistency in cleaning quality. With the development of power automation and robotics, the research on automatic cleaning devices for ceramic post insulators is becoming increasingly important. This article develops an automatic cleaning device for 500 kV porcelain sleeves in the event of power outage maintenance in substations. The device adopts an open cleaning circular ring structure, which can adapt well to the complex environment of the substation site. At the same time, the design of its variable axis length cleaning brush rod can effectively improve the efficiency of cleaning and ensure the consistency of cleaning quality.

In this paper, a type of 5G driven HV electrical device porcelain bushing cleaning robotic arm, which is a kind of mechanical arm with intelligent function, is developed for maintenance of electrical devices in HV substation, precise positioning of the mechanical arm is achieved with the aid of photoelectric switch and the perception of the porcelain bushing position in the space. Furthermore, based on the IoT and edge computing^19^, platform tilt angle is perceived and a kind of leveling control method is proposed. In addition, based on 5G^20,21^ shared base station, the information interaction during the cleaning of Porcelain Bushing is achieved. The field test results indicate that this robotic arm could clean the porcelain bushing effectively with high quality and high security. It enriches the technical meanings of porcelain bushing anti-pollution flashover and improves the state control level of substation equipment.

## Overall design

**Figure 1:**
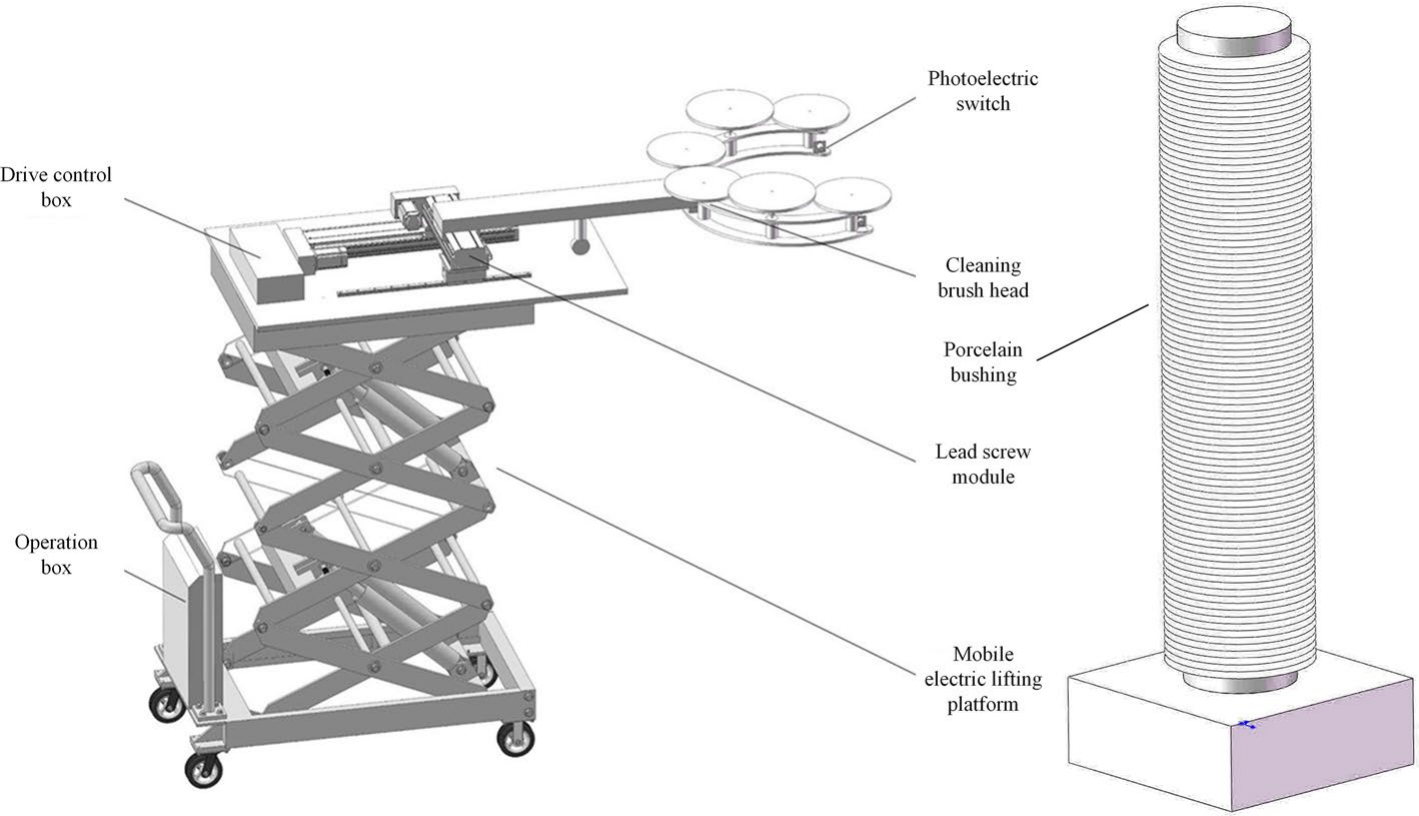
Schematic diagram of robotic arm.

The robotic arm is composed of five components including mobile electric lifting platform, cleaning brush "finger", operation box, drive control box and photoelectric switch and lead screw module. The design scheme of robotic arm is shown in Fig. 1.

The electric lifting platform adopts scissor-lift mechanical structure and hydraulic power transmission to provide the stable support of the whole device. The cleaning brush "finger" is specifically designed which can clean the surface of porcelain bushing. Control button box and industrial control touch screen are set in the operation box, which are used to control the lifting of the electrical lifting platform, the moving speed of the lead screw module and the operating speed of the motor of cleaning brush head. The drive control box realizes the control of the automatic mechanical cleaning arm. Photoelectric switch and lead screw modules are used for precise positioning of the robotic arm, which ensures that the porcelain bushing is centered in the opening ring of the cleaning brush "finger".

## Mechanism design

### Design of cleaning brush "finger"

**Figure 2:**
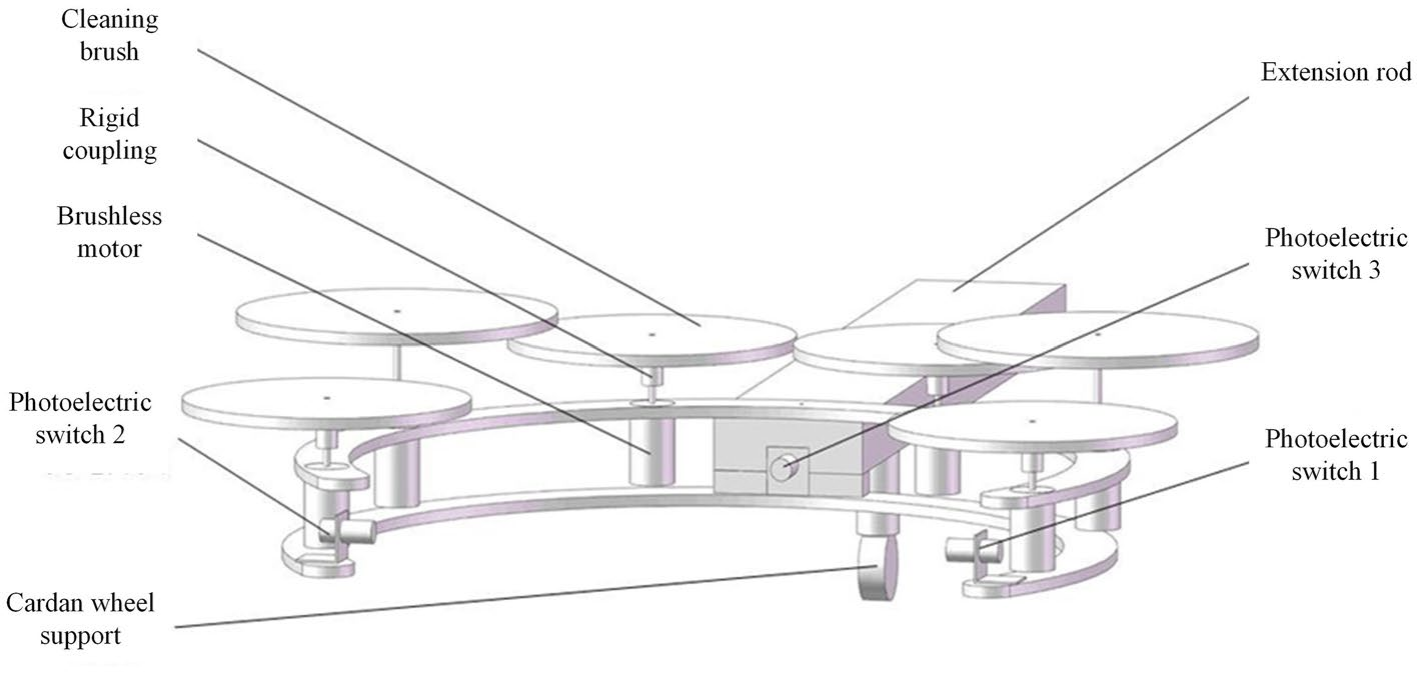
Structure of brush “finger” for robotic arm.

The cleaning brush "finger" is the critical unit of the automatic mechanical cleaning arm. As shown in Fig. 2, the structure of the cleaning brush “finger” with a 120-degree split is proposed. The upper and lower ends of the porcelain bushings of the substation are respectively connected with the base of the inspected device and other devices, so the fixed ring cleaning structure is not suitable for site conditions. In addition, due to the long spatial distance between the porcelain bushing and the ground, it is not convenient to adopt the ring cleaning structure with mechanical opening and closing. The recommended 120-degree-split structure of cleaning brush “finger” can adapt to the complex field environment in the substation lightly. The ringent structure can contribute to approaching the porcelain bushing from different directions.

The cleaning brush "finger" is made of aluminum alloy, on which 6 cleaning motors(brushless motors) and 3 photoelectric switches are installed. The cleaning motor is connected with the cleaning brush "finger" with different coaxial length by a rigid coupling to prevent the collision with adjacent bristles and achieve all-round cleaning within 240 degrees.

In the process of porcelain bushing cleaning, in order to avoid the collision between the electric lifting platform and other devices in the substation, the cleaning brush head is required to extend out of the platform as far as possible. Meanwhile, due to the suspended brush head, the additional torque under the influence of gravity has been produced. Therefore, the structure adopts an extension rod with a cardan wheel support device installed in the middle position. On one hand, the extension of the brush head is achieved, on the other hand, the stability of the structure is improved by cardan wheel support device which brings the significant decrease of the torque impact caused by the self-weight of brush head.

The cleaning brush on the brush "finger" is driven by DC brushless motor, and the speed can be adjusted flexibly depending on the site conditions. DC motor and cleaning brush are connected by rigid coupling, which has good structural stability.

The internal and external double-layer bristles are adopted for the cleaning brush. The length of the outer bristles is longer than that of the outer bristles. The material of the outer bristles is softer than the outer bristles. The two layers of bristles rotate around the same axis to achieve efficient cleaning. According to the practical cleaning experience, there may be friction and collision between adjacent cleaning brush hairs on the same plane, which may affect the actual cleaning effect. Therefore, the structure of brush rod can achieve the dynamic adjustment of adjacent cleaning brush between different working surfaces and avoid the collision between brush hairs. Moreover, the effective cleaning area is enlarged, and the cleaning efficiency is improved.

**Figure 3:**
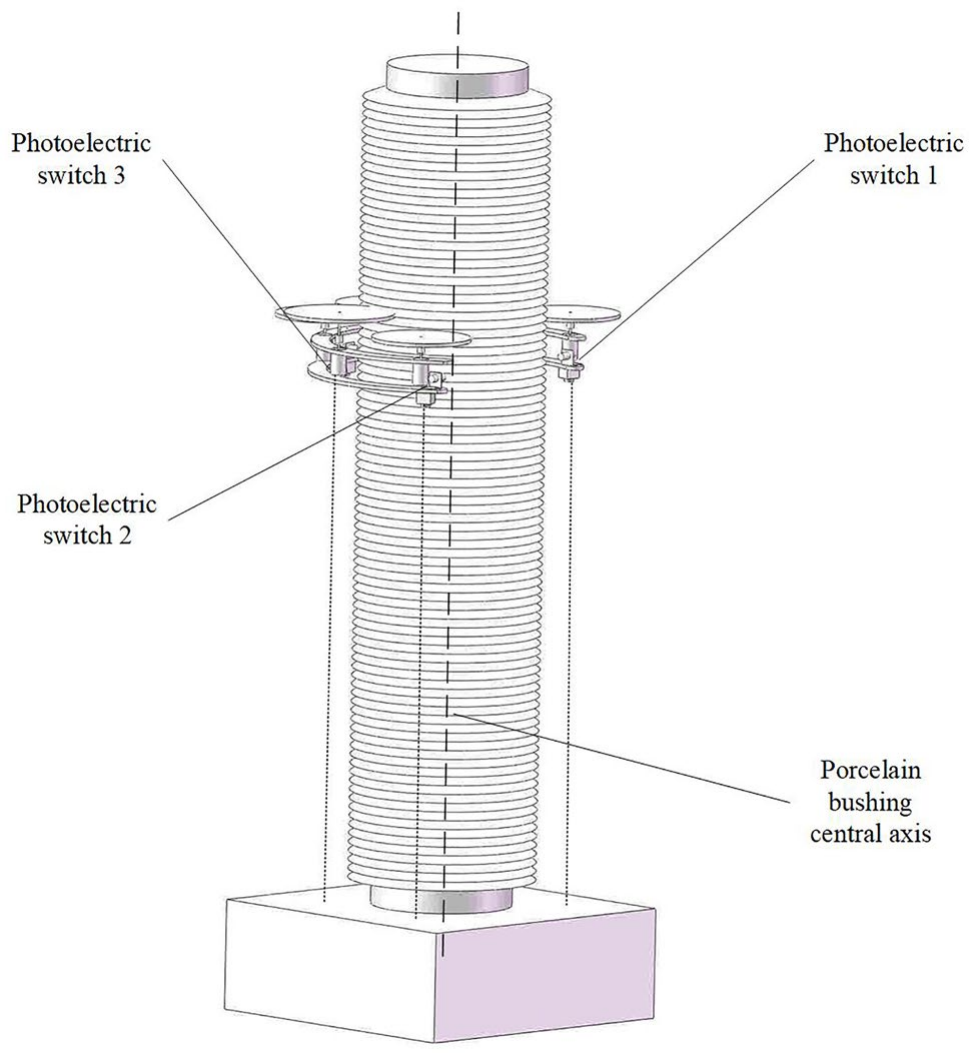
Schematic diagram of cleaning claw and porcelain sleeve.

To express the ideas mentioned in the text more clearly, a two-stage positioning process is used to illustrate, including the schematic diagram of the cleaning claw and porcelain sleeve, as shown in Fig. 3. The specific positioning process can be summarized as the following steps:

1. The operator performs preliminary alignment work, and the insulator cleaning device moves longitudinally to approach the insulator to be cleaned. When the 1st or 2nd photoelectric switch is activated, it enters the first level positioning stage.
2. Based on the operation of the 1st and 2nd photoelectric switches, adjust the horizontal direction of the cleaning brush head until both photoelectric switches operate simultaneously. This indicates that the opening of the cleaning brush head is aligned with the insulator and the first level positioning is completed.
3. Clean the brush head and feed it longitudinally again, further approaching the insulator. When the No. 3 photoelectric switch is activated, it indicates that the insulator to be cleaned is already inside the cleaning brush head and is very close to the No. 3 photoelectric switch at the root of the brush head. At this point, we enter the secondary positioning stage.
4. Based on the pre measured distance information, control the cleaning brush head to retreat to the given distance, ensuring that the insulator is in the center position of the open cleaning brush head, as shown in the following Fig. 3. At this point, the secondary positioning is completed and waiting for the next cleaning process to proceed.

### Photoelectric switch design

**Figure 4:**
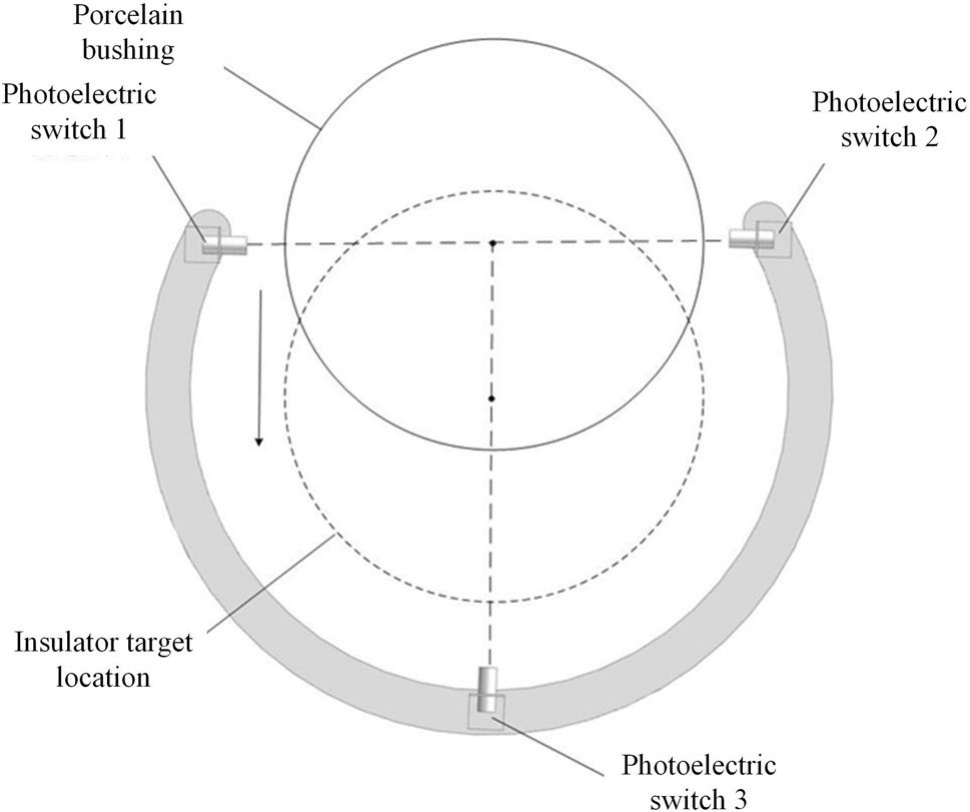
Installation of photoelectric switch of cleaning brush “finger”.

The photoelectric switch is the positioning core of the porcelain bushing automatic mechanical cleaning arm, which is installed at a spacing of 120 degrees on the cleaning brush "finger" ring, as shown in Fig. 4.

No. 1 photoelectric switch and No. 2 photoelectric switch at the opening of ring are installed face to face. Depending on this primary positioning group, the porcelain bushing can be aligned to accurately at the connection center of the opening of ring. No. 3 photoelectric switch is installed at the joint between the end of the cleaning ring and the extension rod, which is positive to the center of the ring as the secondary positioning group. The inductive distance of the photoelectric switch is about 50 mm. According to the feedback of the positioning group, the position of the porcelain sleeve can be detected. The movement of the mechanical cleaning arm is adjusted accordingly, so the porcelain bushing can be located in the center of the opening ring of the cleaning brush “finger”. When the first level positioning action is completed, the porcelain bushing will be located in the connection center of the opening of ring, the lead screw module will drive the cleaning brush head forward, and the porcelain sleeve will entry into the working range of the ring, approaching No. 3 photoelectric switch gradually. When No. 3 photoelectric switch at the joint operates, it demonstrates that the porcelain bushing is already laid in the center of the opening ring, that is, the insulator target location, which represents that the secondary positioning is completed simultaneously. Three photoelectric switches with a spacing of 120 degrees can be positioned in two stages, and they can perform the alignment of the porcelain bushing simply and effectively to prevent the collision risk caused by the deviation of the porcelain bushing position.

## Control method and edge computing

### Control system design based on S7-200 PLC

**Figure 5:**
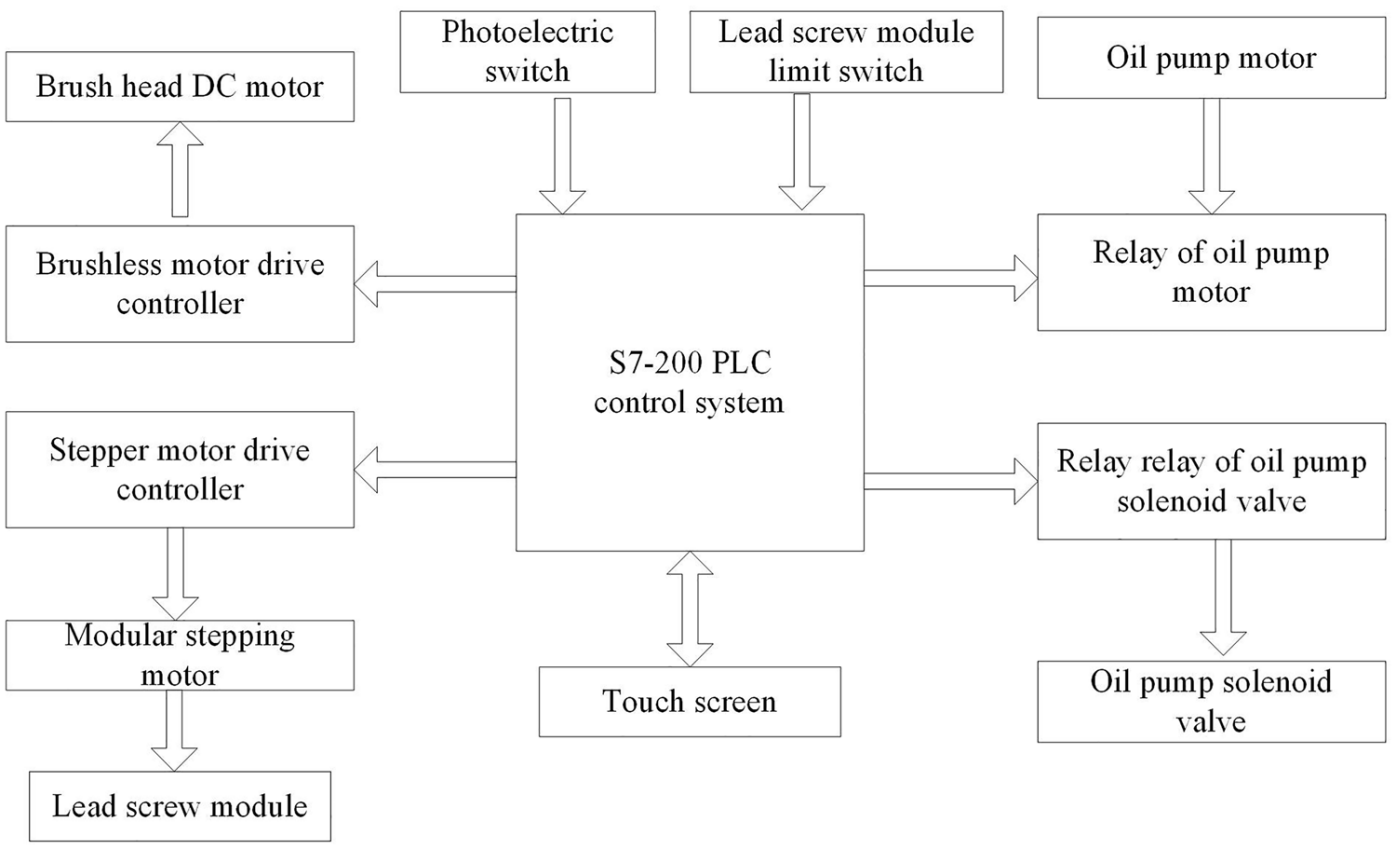
Schematic diagram of device system.

Figure 5 is the schematic diagram of the system. S7-200 PLC is the control unit of the whole cleaning robotic arm, which controls the action of each actuator of the cleaning robotic arm by receiving the signal from the sensors. The position of the porcelain bushing is judged by the photoelectric switch. After receiving the special signal, a high-speed pulse is sent out by the PLC to drive the stepper motor driver, so that the lead screw module can be adjusted to the appropriate position. The photoelectric switch and the module limit switch judge the position of the target porcelain bushing accurately according to the sensor feedback. The relay is used to height adjustment of the lifting platform by controlling the oil pump motor and oil pump solenoid valve.

The upper device adopts industrial touch screen to communicate with S7-200 PLC for motion control and parameter setting.

### Automatic leveling control mechanism

**Figure 6:**
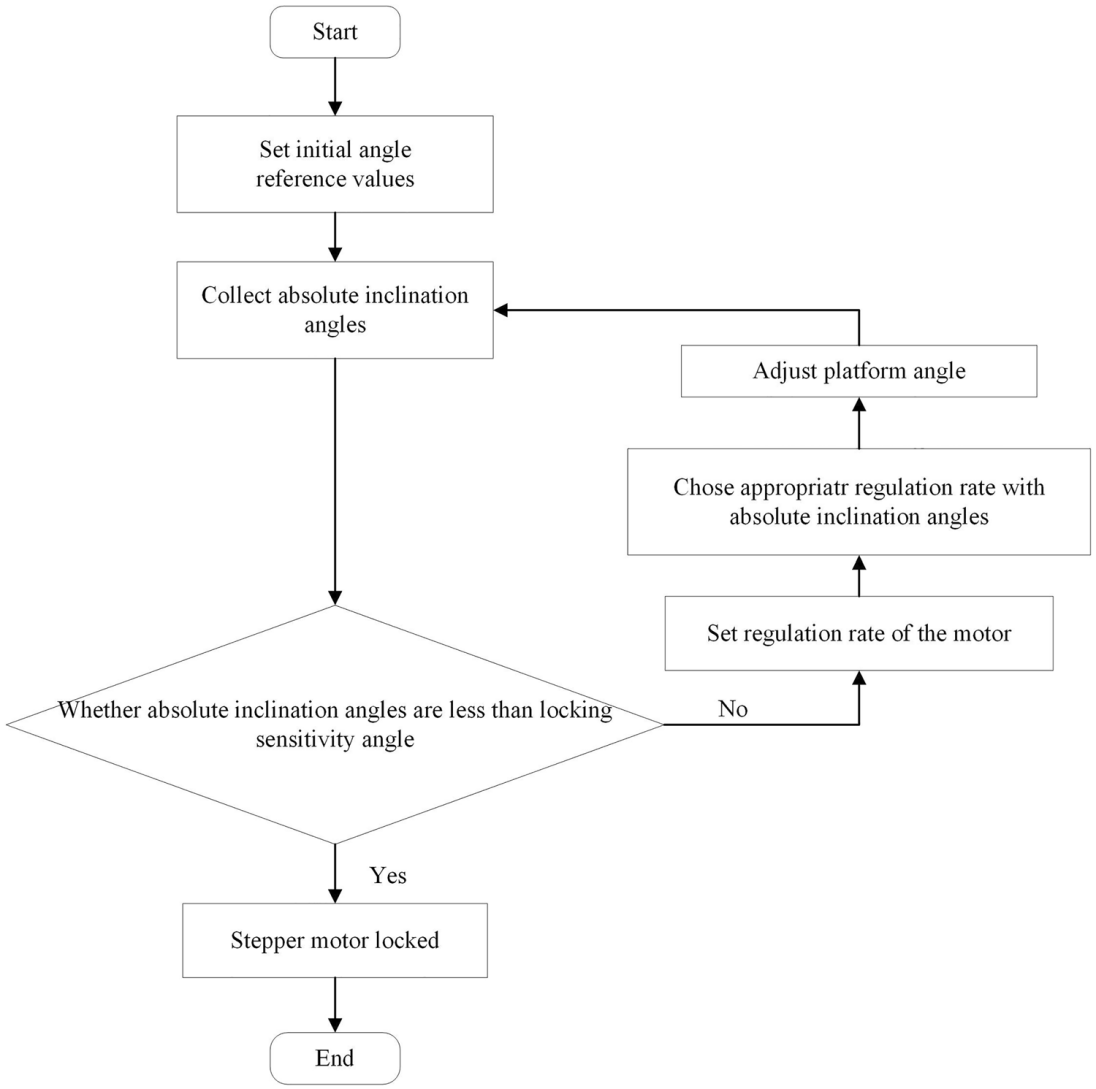
Control flow chart of real-time angle feedback control method.

In order to ensure that the cleaning brush “finger” ring will not collide with the porcelain sleeve in the process of leveling of the electric lifting platform, it is required that the angle of the lifting platform is within the allowable error range, and the angle should be adjusted automatically with edge computing when the angle is out of range. The control flow chart is shown in Fig. 6.

The platform leveling of this arm adopts the real-time angle-feedback control method, and utilizes three groups of gyroscopes installed in the driving control box to collect the data of tilt angle of the platform under the working condition. Before acquiring angle, three groups of initial angle reference values are set in advance. $\beta_{i,k}^{X}$*,*$\beta_{i,k}^{Y}$*,*$\beta_{i,k}^{Z}$ (*i* ∈ *N, k* = 1*,* 2*,* 3*,* 4), the absolute inclination angles of the load surface and the horizontal plane in three dimensions (*x, y, z*) is collected respectively at the rate of 40 times/second. The absolute inclination for control is calculated as follows.

1 $$\left\{ {\begin{array}{*{20}l} {\alpha_{i}^{X} = \frac{1}{4}\sum\limits_{k = 1}^{4} {\beta_{i,k}^{X} } } \hfill & {i \in N} \hfill \\ {\alpha_{i}^{Y} = \frac{1}{4}\sum\limits_{k = 1}^{4} {\beta_{i,k}^{Y} } } \hfill & {i \in N} \hfill \\ {\alpha_{i}^{Z} = \frac{1}{4}\sum\limits_{k = 1}^{4} {\beta_{i,k}^{Z} } } \hfill & {i \in N} \hfill \\ \end{array} } \right.$$

$$\user2{\rm A}_{i}^{{}} = \left( {\begin{array}{*{20}c} {\alpha_{i}^{X} ,} & {\alpha_{i}^{Y} ,} & {\alpha_{i}^{Z} } \\ \end{array} } \right)$$, which represents the absolute inclination of the platform in three dimensions.

In order to level the arm, the ideal value of adjustment rate in three dimensions ${\varvec{R}}_{j}^{{}}$, which is shown as $${\varvec{R}}_{j}^{{}} = \left( {\begin{array}{*{20}c} {r_{j}^{X} ,} & {r_{j}^{Y} ,} & {r_{j}^{Z} } \\ \end{array} } \right) \, j = 1, \, 2$$, is further introduced. According to the absolute tilt angle $\user2{\rm A}_{i}^{{}}$, the rotation speed of the stepping motor located at the bottom leg of the electric lifting platform is dynamically adjusted.

To reduce the influence of accumulated error, when the arm moves, the reference values of angle in each dimension are reset every 10 s, and when the movement of the arm is aborted and restarted, the reference value of angle should be reset.

After the angle data α of each group of gyroscopes is detected, the stepping motor corresponding to the bottom leg of the platform is activated to achieve the angle control of the platform. When ***A***_***i***_ is in a neighborhood of the origin of 3D space, namely $\alpha_{i}^{X} \in \left[ { - \varepsilon^{X} ,\varepsilon^{X} } \right]$, $i \in N$; $\alpha_{i}^{Y} \in \left[ { - \varepsilon^{Y} ,\varepsilon^{Y} } \right]$, $i \in N$;$\alpha_{i}^{Z} \in \left[ { - \varepsilon^{Z} ,\varepsilon^{Z} } \right]$,$i \in N$; the stepping motor is locked. It should be noted that $\varepsilon^{X}$, $\varepsilon^{Y}$, $\varepsilon^{Z}$ are the locked sensitivity angles of each dimension. It is recommended that the locked sensitivity angle of each dimension is *θ*_0_ to simplify the discussion.

The real-time angle-feedback control method used in this arm is composed of two practical control strategies including threshold control and flexible control, which can be selected and switched through the Human Machine Interface (HMI).

The threshold control strategy with x-axis leveling control is shown as follow. When the gyroscope detects that $\alpha_{i}^{X}$ exceeds [− $\theta_{1}^{{}}$,$\theta_{1}^{{}}$], the speed of the stepping motor is adjusted at the rate of $r_{1}^{X}$. When $\alpha_{i}^{X}$ returns to [− $\theta_{1}^{{}}$,$\theta_{1}^{{}}$] and is not in the locked range, the speed of the stepping motor slows down, which represents that the speed is adjusted at a rate of $r_{2}^{X}$.

Under the threshold control strategy, the x-axis regulation rate at time *i* is shown as

2 $$r_{{}}^{X} = \left\{ {\begin{array}{*{20}l} {r_{1}^{X} } \hfill & {\alpha_{i}^{X} \notin \left[ {\begin{array}{*{20}c} { - \theta_{1}^{{}} } & {\theta_{1}^{{}} } \\ \end{array} } \right]} \hfill \\ {r_{2}^{X} } \hfill & {\alpha_{i}^{X} \in \left[ {\begin{array}{*{20}c} { - \theta_{1}^{{}} } & { - \theta_{0}^{{}} } \\ \end{array} } \right] \cup \left[ {\begin{array}{*{20}c} {\theta_{0}^{{}} } & {\theta_{1}^{{}} } \\ \end{array} } \right]} \hfill \\ 0 \hfill & {\alpha_{i}^{X} \in \left[ {\begin{array}{*{20}c} { - \theta_{0}^{{}} } & {\theta_{0}^{{}} } \\ \end{array} } \right]} \hfill \\ \end{array} } \right.$$

where $$r_{1}^{X} > r_{2}^{X} , \, \theta_{1} > \theta_{0}$$.

The flexible control strategy refers to the flexible control of the leg stepping motor by introducing the regime switching function. Expression of the regime switching function proposed by Chen et al.^8^ taking the x-axis as an example is as follow:

3 $$F(\alpha_{i}^{X} ;\Delta ,\gamma ) = \frac{1}{{1 + \exp ( - \gamma (\left| {\alpha_{i}^{X} } \right| - \Delta ))}}$$

where $$\Delta$$ is the threshold value; $$\gamma$$ is the conversion speed parameter, which is generally taken as a positive integer and satisfies that $$\gamma$$ is further greater than $${\raise0.7ex\hbox{$1$} \!\mathord{\left/ {\vphantom {1 \Delta }}\right.\kern-0pt} \!\lower0.7ex\hbox{$\Delta $}}$$. Keeping $$\left| {\alpha_{i}^{X} } \right| = 0$$,$$F \to 0$$;$$\left| {\alpha_{i}^{X} } \right| = \Delta$$,$$F \to 0.5$$;$$\left| {\alpha_{i}^{X} } \right| \to \infty$$,$$F{ = 1}$$. $$\Delta$$ is the threshold parameter.

When the gyroscope detects that $\alpha_{i}^{X}$ exceeds [− $\theta_{1}^{{}}$,$\theta_{1}^{{}}$], speed regulation is transited to $r_{1}^{X}$ by regime switching function (3). When $\alpha_{i}^{X}$ returns to [− $\theta_{1}^{{}}$,$\theta_{1}^{{}}$] and the motor is not locked, the speed of stepping motor slows down and the angle of platform is adjusted softly.

According to the flexible control strategy, based on the regime switching function, the regulation rate at time i is as shown

4 $$r_{{}}^{X} = \left\{ \begin{gathered} \begin{array}{*{20}c} {r_{2}^{X} + \frac{{r_{1}^{X} - r_{2}^{X} }}{{1 + \exp ( - \gamma (\left| {\alpha_{i}^{{}} } \right| - \theta_{1}^{{}} ))}}} & {\alpha_{i}^{X} \notin \left( {\begin{array}{*{20}c} { - \theta_{0}^{{}} } & {\theta_{0}^{{}} } \\ \end{array} } \right)} \\ \end{array} \hfill \\ \begin{array}{*{20}c} 0 & {\alpha_{i}^{X} \in \left( {\begin{array}{*{20}c} { - \theta_{0}^{{}} } & {\theta_{0}^{{}} } \\ \end{array} } \right)} \\ \end{array} \hfill \\ \end{gathered} \right.$$

where $r_{1}^{X} > r_{2}^{X}$. The recommended threshold value $\Delta$ is *θ*_0_, and the recommended range of the conversion speed parameter* γ* is [400, 600].

The threshold control strategy achieves the real-time control of absolute tilt angle, which has the advantage of fast response time (within 500 ms) while the adjustment accuracy is low. The advantage of the flexible control strategy is that the adjustment is more accurate, the adjustment process is smoother, and the overshoot is smaller, which can ensure that the inclination of the load surface can be adjusted to within *θ*1 at a suitable speed. Two strategies are selected on the basis of the practical restrictions of the site. Generally, the two strategies can meet the practical needs in speed adjustment. According to practical maintenance experience in the HV substation, the recommended value of *θ*_0_ is 2°. Moreover, the value of *θ*_1_ should meet (5) as follows:

5 $$\theta_{1}^{{}} \ge k\theta_{0}^{{}} + \theta_{\min }^{{}}$$

where margin parameter *k* is more than 2. and *θ*_min_ is the minimum angle that can be identified. The recommended value of *θ*_1_ is 5°.

**Figure 7:**
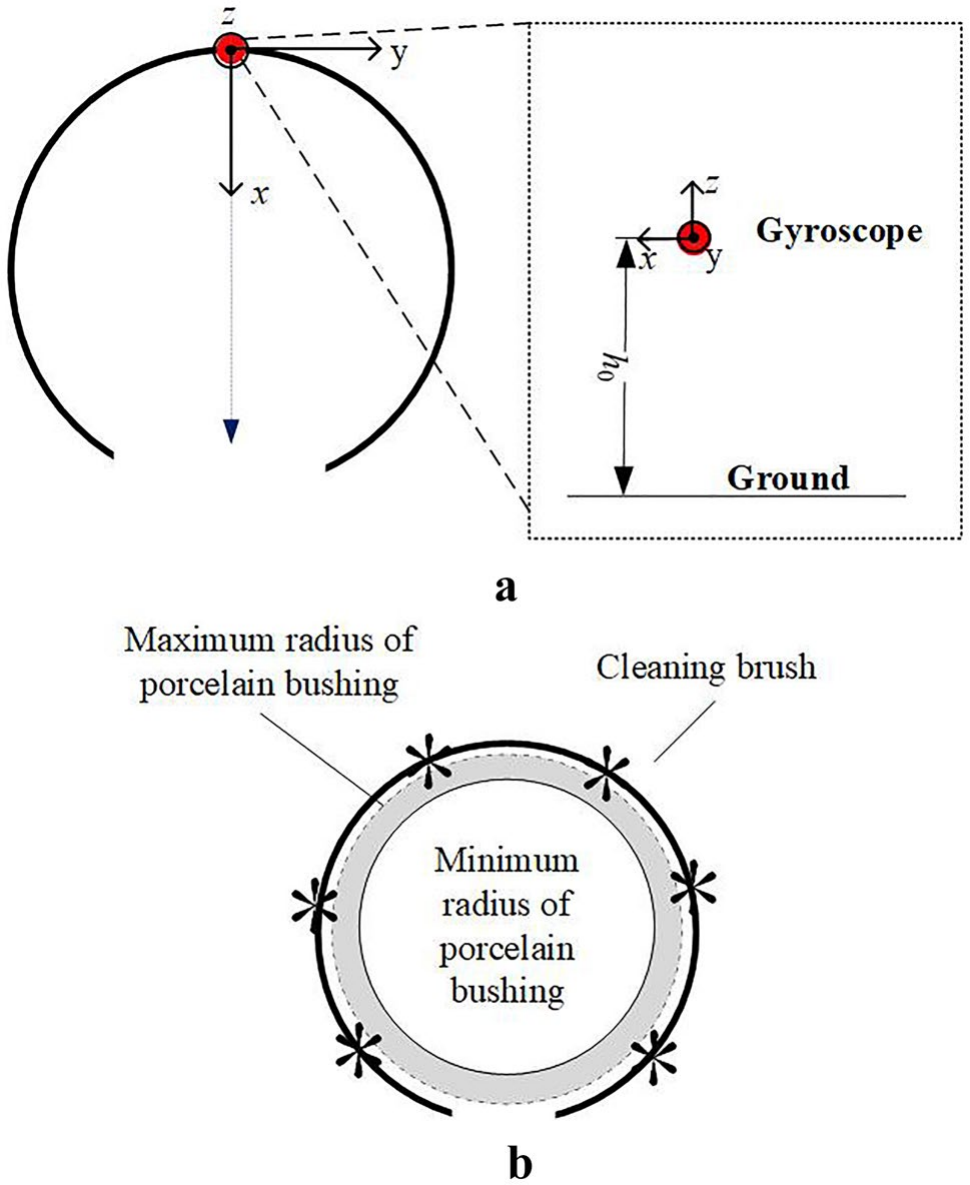
Schematic diagram of coordinate system description. (**a**) Coordinate system diagram. (**b**) Coordinate system diagram.

To illustrate the requirements of the coordinate system more clearly, use Fig. 7 for illustration. Establish an Oxyz coordinate system as shown in Fig. 7, with the ground as the reference plane and the projection point of sensor 3 as the origin, to describe the spatial position information.

The real-time values of x-axis, y-axis, z-axis regulating speed and torque sensor are collected by intelligent maintenance terminal. Afterwards, the regulating speed vector is synthesized and the real-time map of regulating speed vector is drawn utilizing the edge computing. Ultimately the optimized control strategy is obtained. All these valuable information is sent back to the monitoring system by 5G network.

### Perception technology of charged area based on UWB

Considering the complex electrical environment in the 500 kV substation, when the automatic cleaning robotic arm works, it is necessary to keep sufficient distance between the arm and the electrical devices in HV substation to ensure the safety of devices and the safety of personnel.

Owing to edge computing, a safe-distance control method and location method based on UWB location technology is presented. Adopting two-way time of flight method for ranging is the principle of UWB ranging. Base station transmits request pulse signal at *T*_*a*1_, and tag receives request signal at time *T*_*b*1_ and transmits a response signal at time *T*_*b*2_. Base station receives the response signal at time *T*_*a*2_, and then the distance *R* between base station and tag can be calculated by (6).

6 $$R = C \times \left[ {\left( {T_{a2} - T_{a1} } \right) - \left( {T_{b2} - T_{b1} } \right)} \right]$$

where *C* is the speed of light.

**Figure 8:**
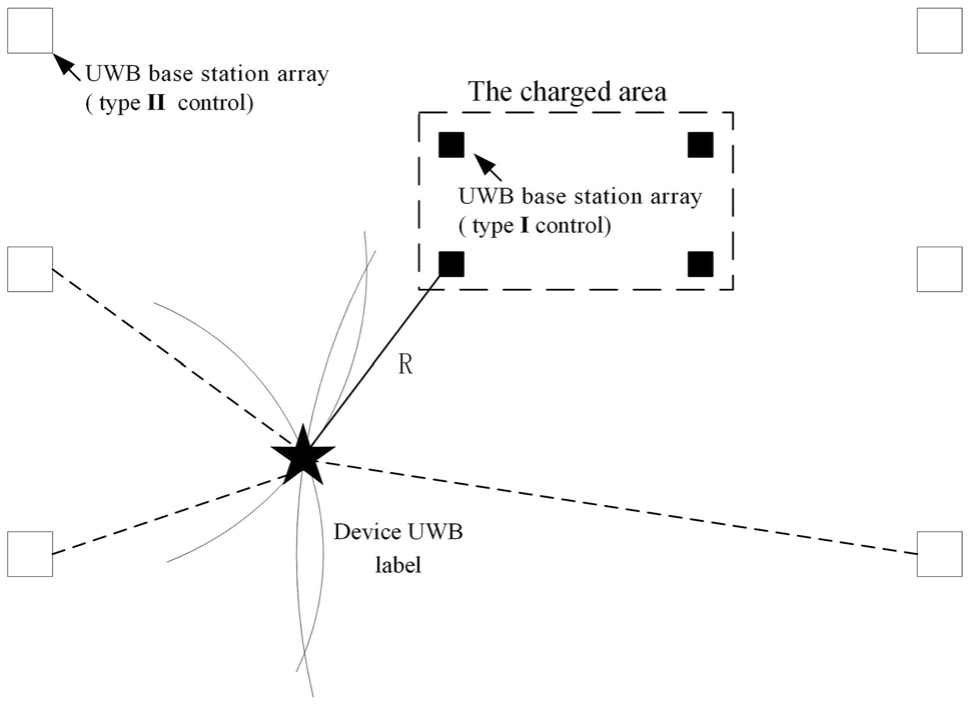
Two types of location method.

The dual safe distance control method of mobile UWB base station array and standing UWB base station array is employed to perform charged area sensing. This method can be divided into two types (type I control and type II control), as shown in Fig. 8.

#### Safe distance control based on mobile UWB base station array (type I control)

A mobile light UWB base station array is arranged around the outage area, and a UWB label is arranged on the automatic mechanical cleaning arm to perform the dynamic calculation of the distance between the arm and the charged area. When the distance *R* between any light UWB base station and the label is less than the low warning threshold (LWT), an audible and visual alarm is triggered. When the distance *R* between the base station and the label is less than the high warning threshold (HWT), the arm is locked and the power supply is interrupted forcibly.

#### Safe distance control based on standing UWB base station array (type II control).

In order to monitor the position of equipment and workers in the substation and achieve better professional management, a standing UWB base station array is arranged in the primary site of the substation. Based on the distance measurement of the UWB label loaded on the automatic sweeping arm, the distance information between the device label and different standing UWB base stations can be obtained. Using more than three distance information, the specific position of the automatic sweeping arm in the working area of the substation can be determined by the intersecting circle principle, and the relevant position information and sweeping information can be transmitted back to the main control room of the substation by 5G network. Therefore, a real-time charged-area warning map can be drawn based on the returned data.

Owing to edge computing, the small-scale positioning of the working area of the arm is achieved using the type II control, which isolates the charged area near the work site and ensures the safety of the equipment and the safety of personnel during the cleaning. The efficient control of personnel movement track can be obtained and the major accidents such as inrush of charged bay are effectively prevented by means of type I control.

This article proposes a secure distance control and positioning method based on UWB positioning technology. Ultra wideband (UWB) technology is a radio technology based on the IEEE802.15.4a and 802.15.4z standards, which can accurately measure the flight time of radio signals, thereby achieving centimeter precision distance/position measurement. Unlike other positioning technologies such as Bluetooth and WiFi, the inherent physical characteristics of UWB RF signals have defined UWB technology from the beginning: achieving real-time, ultra precise, and ultra reliable positioning and communication. UWB positioning technology has high positioning accuracy and is not affected by harsh conditions such as dust, rain, and snow. It can be used under high voltage and strong magnetic field conditions. At the same time, its position refresh frequency is high, and it can send the target object position to the management platform for presentation without delay.

### Simulation

**Figure 9:**
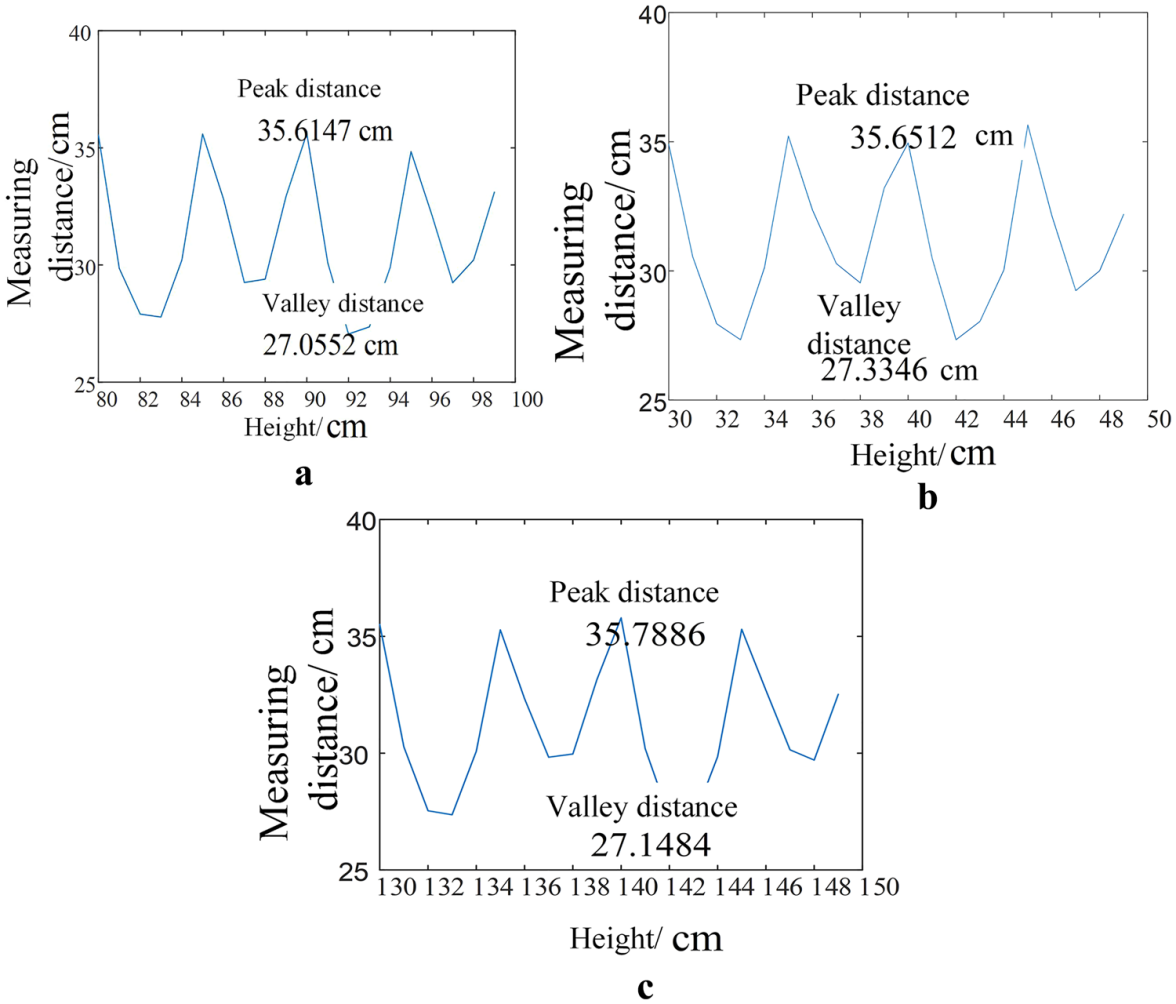
Measuring results sensors during cleaning. (**a**) Measuring result of sensor No. 1. (**b**) Measuring result of sensor No. 2. (**c**) Measuring result of sensor No. 3.

**Figure 10:**
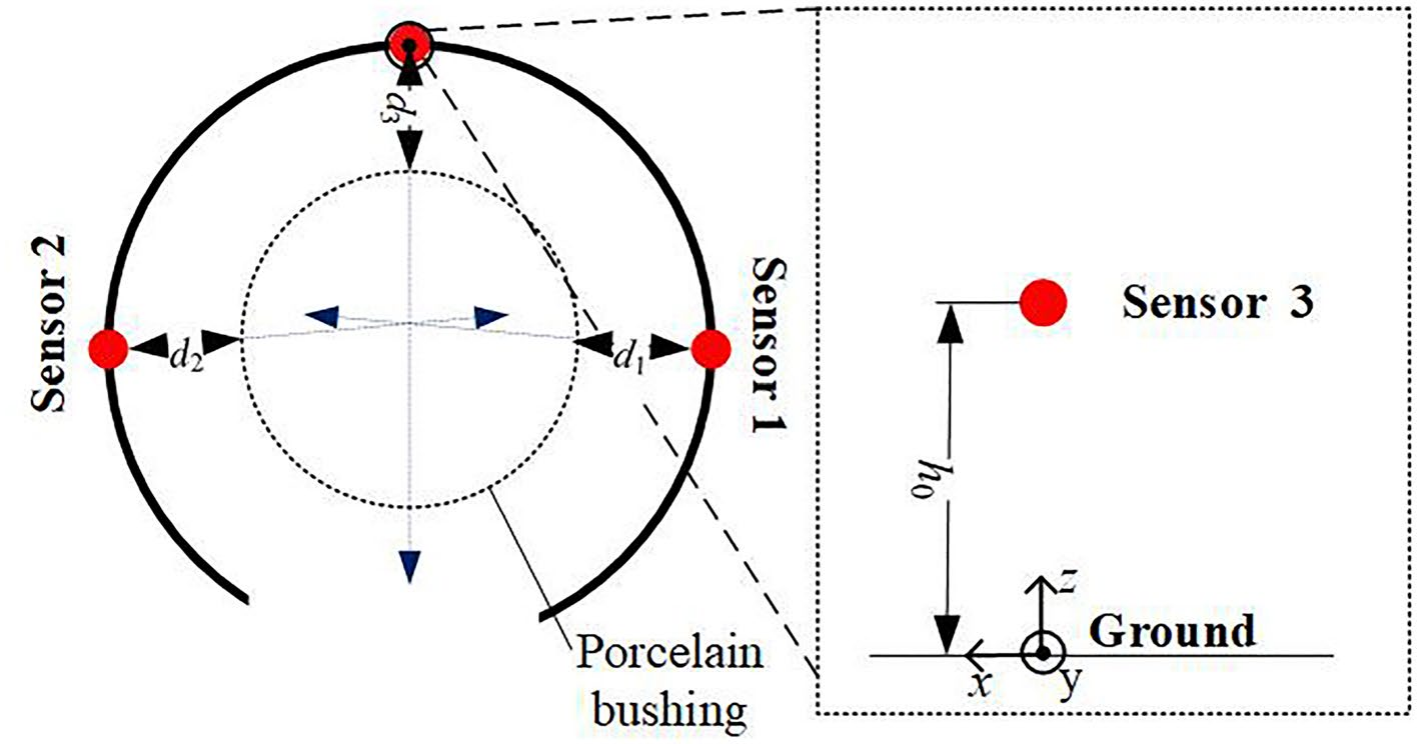
Distance simulation diagram.

**Figure 11:**
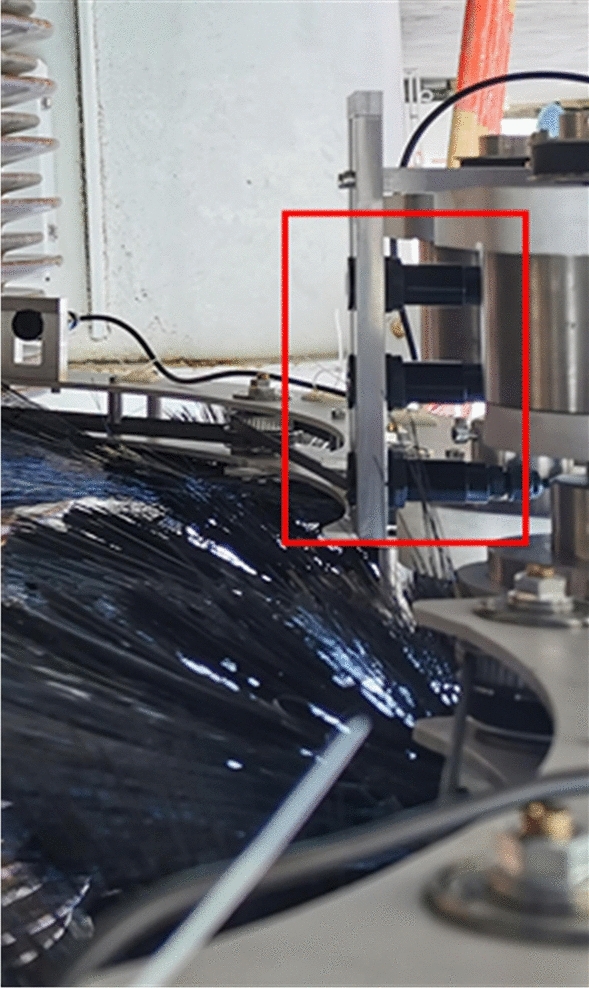
Laser sensor diagram.

The basic design requirement of the cleaning device is to realize the safe and reliable cleaning of the Porcelain Bushing. Operation reliability requires that the device can accurately identify the Porcelain Bushing and ensure that it is basically in the center of the cleaning brush. However, operation safety requires that the device does not collide with the body of Porcelain Bushing during the process of hugging and cleaning. Since the low-cost two-stage automatic orientation method is used in this paper to realize the alignment of the Porcelain Bushing by measuring distance. In order to verify its reliability and safety, a 120° distributed laser sensor (No. 1, No. 2, No. 3) is installed at the position of the cleaning brush "finger" of the device. Whether the Porcelain Bushing is in the center of the cleaning brush "finger" is judged by the distance measurement result fed back during the cleaning process, as shown in Fig. 9. Table 1 is the record of the ranging results. The data demonstrates that the two-stage simple and effective method proposed in this paper can effectively realize the alignment of the Porcelain Bushing and ensure the safety and stability of the subsequent cleaning work. The coordinate system diagram in sensor positioning is shown in Fig. 10 below. The laser sensor experiment is shown in Fig. 11.

**Table 1:** Detection results of feature.

Sensor number	1	2	3
Peak distance (m)	0.356	0.356	0.358
Valley distance (m)	0.271	0.273	0.271

**Figure 12:**
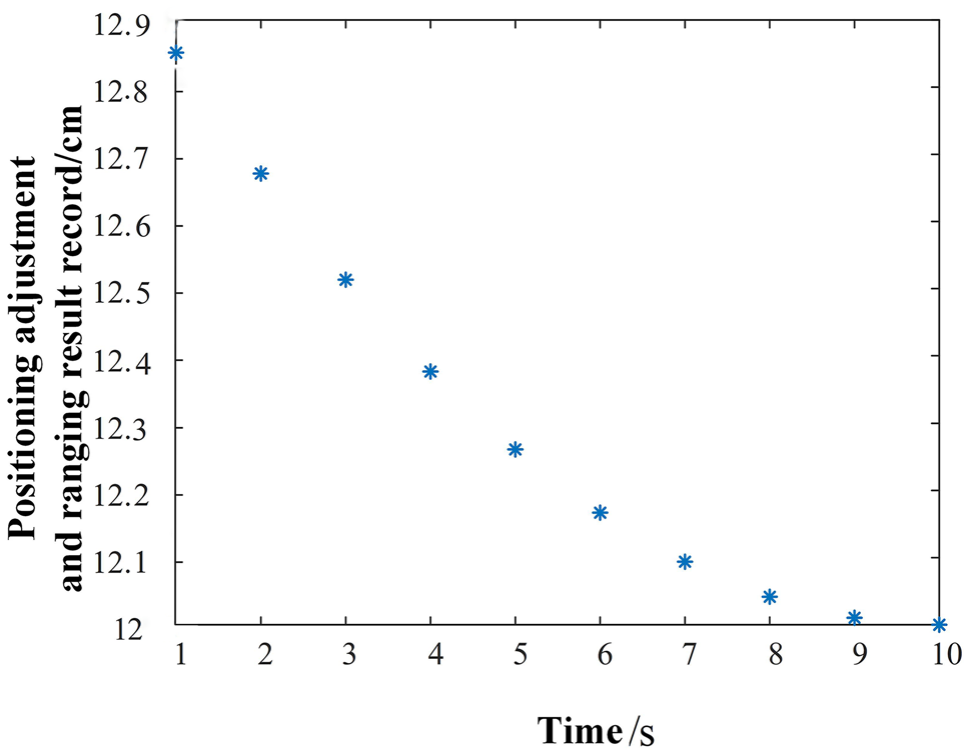
Positioning adjustment and measuring results.

After actual experiments, the key data of distance measurement during the adjustment process of the cleaning brush are shown in Fig. 12. The robotic arm smoothly approaches the Porcelain Bushing without exceeding the preset safety distance (0.05 m), which ensures the reliability and safety of subsequent cleaning. Further, the specific action speed of the cleaning robot can be flexibly adjusted according to the preset motion parameters, and the whole cleaning process can be ensured with good stability and consistency through multiple debugging.

## Construction of 5G shared base station

The intensity of signal can be weakened in special domain in switch yard by the strong electromagnetic interference in HV substation. The transmission of the edge computing results got from switch yard will be seriously affected and the control performance will be poor. To solve this problem, 5G network can be used as the physical basis for the robotic arm to transmit the edge computing results to the monitoring and control system.

Although China is racing to build 5G networks, the construction of 5G base station still seems a long way off. Accelerating the distribution of 5G base stations near the substation is conducive to the advanced technology based on 5G, which may improve the intelligent maintenance level of the substation. In this paper, a new idea of 5G shared base station construction is proposed.

The roof of some substations will be opened to 5G operators to arrange 5G antennas, so that there will no longer exist the difficulty of base station positioning.

Massive MIMO technology is popularly applied in 5G base station and the demand of power supply is 2 or 3 times more than the demand in 5G base station. Power supply is a huge challenge for the 5G operators. In 5G shared base station, since highly reliable power transformed from HV substation can supply dozens of 5G base station, uninterrupted power supply to communication equipment of 5G base station is guaranteed.

The inspection, operation and maintenance of 5G base station should be included in the overall maintenance system of the substation to reduce the maintenance costs of operators significantly. For example, the inspection robot in the 500 kV HV substation inspect all the devices for 3 times one day, so the inspection cost of the equipments in 5G base station is absolutely reduced. Therefore, though it will cost much on the construction of 5G base station, the benefit of using 5G is still considerable.

According to the above methods, a 500 kV substation has been rebuilt as a 5G shared base station in advance, and 5G information transmission test and network storm test using simulation data has been carried out. Before the field test of the robotic arm, the technical preparation of 5G information interaction has been completed.

## Field test

According to the porcelain structure of a typical 220 kV Circuit Breaker in 500 kV substation, corresponding structural parameters of the porcelain cleaning robotic arm are set, and the prototype of the robotic arm is developed. The parameters of the robotic arm are summarized in Table 2.

**Table 2:** Parameters of the robotic arm.

Items	Value
Weight of the cleaning brush "finger" and the lead screw module	55 kg
Weight of the robotic arm	520 kg
The length of the extension rod of the cleaning brush "finger"	0.9 m
The number of photoelectric switches	3
Maximum extension height	6.6 m

**Figure 13:**
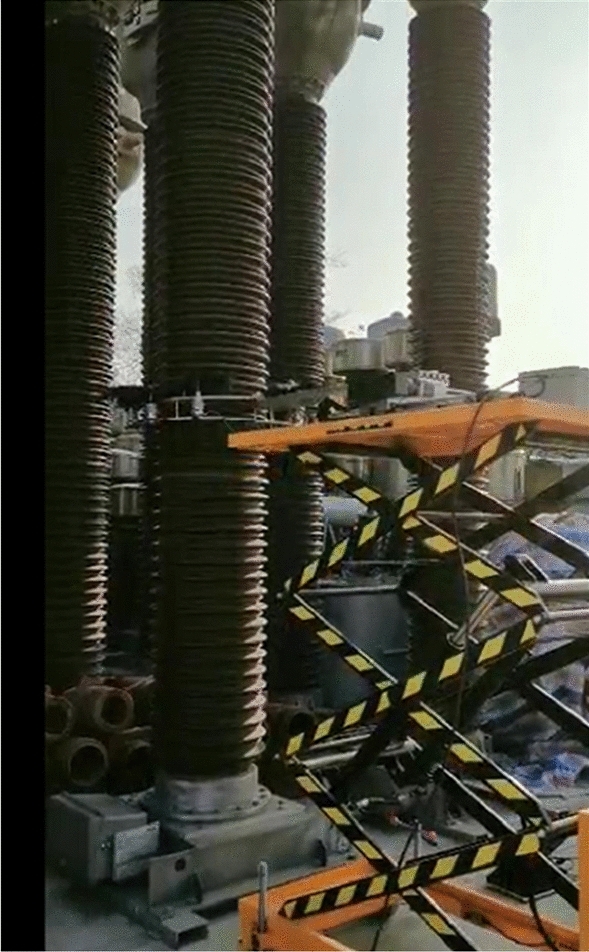
Device on-site testing diagram.

After the preliminary commissioning in the laboratory, cleaning test on porcelain bushing of CB has been performed in the power grid of Nanjing, as shown in Fig. 13.

After the preliminary commissioning in the laboratory, cleaning test on porcelain bushing of CB has been performed in the power grid of Nanjing.

When the robotic arm is close to the CB under test, the platform is leveled according to the established control strategy to ensure that the level of the lifting platform is always in a balanced state, and the adjustment need to be completed within 0.5 s.

When the robotic arm reached the designated test position, the arm starts to clean the CB porcelain bushing. All sensors operate normally. It takes 30 s to clean a 4.8 m long 500 kV porcelain bushing. The field test shows that the initial positioning time of workers before cleaning is more discrete which is 20–75 s denoting that the total cleaning time is 50–105 s, which improves the efficiency of manual cleaning significantly.

During the dynamic and static process, the robotic arm correctly perceives the surrounding electriferous area and effectively alarm or cut off the power supply, and the sensitivity reaches the design expectation. The robotic arm achieves platform leveling according to the established control strategy, and the single leveling time is about 300 ms (threshold control strategy) to 650 ms (flexible control strategy). Edge computing is employed to deal with the sense information. Based on the result of edge computing, the control strategy drives the arm to clean the CB porcelain bushing. Moreover, the result of edge computing is transmitted to the monitoring center by 5G technology. The performance of the robotic arm are summarized in Table 3. By comparison, The performance of conventional manual methods are shown in Table 3 as well.

**Table 3:** Performance comparison of the robotic arm and conventional method.

Items	Value
Robotic arm	
The total cleaning time	30–40 s
Platform leveling time (threshold control strategy)	300 ms
Platform leveling time (flexible control strategy)	650 ms
Conventional methods	
The total cleaning time (skilled worker)	12–20 min
The total cleaning time (average worker)	15–30 min

According to Table 3, the robotic arm shows higher efficiency than conventional methods.

In the 5G shared base station, the signal is distortionless during the process of field test, which effectively realizes the fast transmission of related information.

In conclusion, the main performance targets of the porcelain cleaning robotic arm have been achieved and even exceeded the design expectations.

## Conclusion

In this paper, the cleaning robotic arm for porcelain bushing of high-voltage devices in substation is proposed. In order to adapt to the complex environment of the substation, the opening cleaning ring structure is adopted in mechanism design. The edge computing is used in the control system. A new platform leveling control strategy and the perception method of the charged area based on UWB are proposed. Moreover, the technical framework of the construction of 5G shared base station is introduced. The field test results show that the robotic arm has a good ability of porcelain bushing cleaning. The technical measures against pollution flashover of porcelain bushing are enriched. The state control level of substation equipment is improved, and important guidance for the future research of intelligent operation and inspection is provided.

In the future, structure of the brush head of the porcelain cleaning robotic arm will be further optimized and the adaptability of different size porcelain should be enhanced. Along with the edge computing and 5G technology, the positioning accuracy and control efficiency of the arm can be further improved. Besides, the integration of the arm, its drive control system and upper computer software also need to be improved.

## Data Availability

The datasets generated and/or analysed during the current study are not publicly available due to the need to protect sensitive information, but are available from the corresponding author on reasonable request.
