# Author Correction: Design of robotic arm for the porcelain bushing in substation

**DOI:** 10.1038/s41598-024-65253-4

**Published:** 2024-06-21

**Authors:** Hao Chen, Wei Han, Weilun Xu, Zongyao Tang, Yini Chen, Peng Xu, Zhaoxing Ma

**Affiliations:** 1 State Grid Jiangsu Electric Power Co. Ltd. Nanjing Power Supply Branch, Nanjing, 211102 China; 2 Electric Power Research Institute of State Grid Henan Electric Power Company, Zhengzhou, 450052 China; 3 State Grid Jiangsu Electric Power Co. Ltd. EHV Branch, Nanjing, 211100 China; 4 State Key Laboratory of Technology and Equipment for Defense Against Power System Operational Risks, NARI Group Co., Ltd., Nanjing, China; 5 School of Information and Control Engineering, Qingdao University of Technology, Qingdao, China

Correction to: *Scientific Reports* 10.1038/s41598-024-58443-7, published online 02 April 2024

The original version of this Article contained errors.

In the original version of this Article, the Affiliation list was incorrect.

- Affiliation 1: ‘State Grid Jiangsu Electric Power Co., Ltd. Nanjing Power Supply Branch, Nanjing, China.’
- Affiliation 2: ‘State Key Laboratory of Technology and Equipment for Defense against Power System Operational Risks, NARI Group Co., Ltd., Nanjing, China.’
- Affiliation 3: ‘School of Information and Control Engineering, Qingdao University of Technology, Qingdao, China.’

The correct Affiliations are listed below:

- Affiliation 1: ‘State Grid Jiangsu Electric Power Co. Ltd. Nanjing Power Supply Branch, Nanjing 211102, China.’
- Affiliation 2: ‘Electric Power Research Institute of State Grid Henan Electric Power Company, Zhengzhou 450052, China.’
- Affiliation 3: ‘State Grid Jiangsu Electric Power Co. Ltd. EHV Branch, Nanjing 211100, China.’
- Affiliation 4: ‘State Key Laboratory of Technology and Equipment for Defense against Power System Operational Risks, NARI Group Co., Ltd. Nanjing, China.’
- Affiliation 5: ‘School of information and control engineering, Qingdao University of Technology, Qingdao, China.’

In addition, the authors Wei Han, Zongyao Tang, Peng Xu and Zhaoxing Ma were incorrectly affiliated. The correct Affiliations are listed below:

Wei Han

- ‘Electric Power Research Institute of State Grid Henan Electric Power Company, Zhengzhou 450052, China.’

Zongyao Tang

- ‘State Grid Jiangsu Electric Power Co.Ltd. EHV Branch, Nanjing 211100, China.’

Peng Xu

- ‘State Grid Jiangsu Electric Power Co. Ltd. EHV Branch, Nanjing 211100, China.’

Zhaoxing Ma

- ‘State Key Laboratory of Technology and Equipment for Defense against Power System Operational Risks, NARI Group Co., Ltd. Nanjing, China.’
- ‘School of information and control engineering, Qingdao University of Technology, Qingdao, China.’

The original Article has been corrected.

